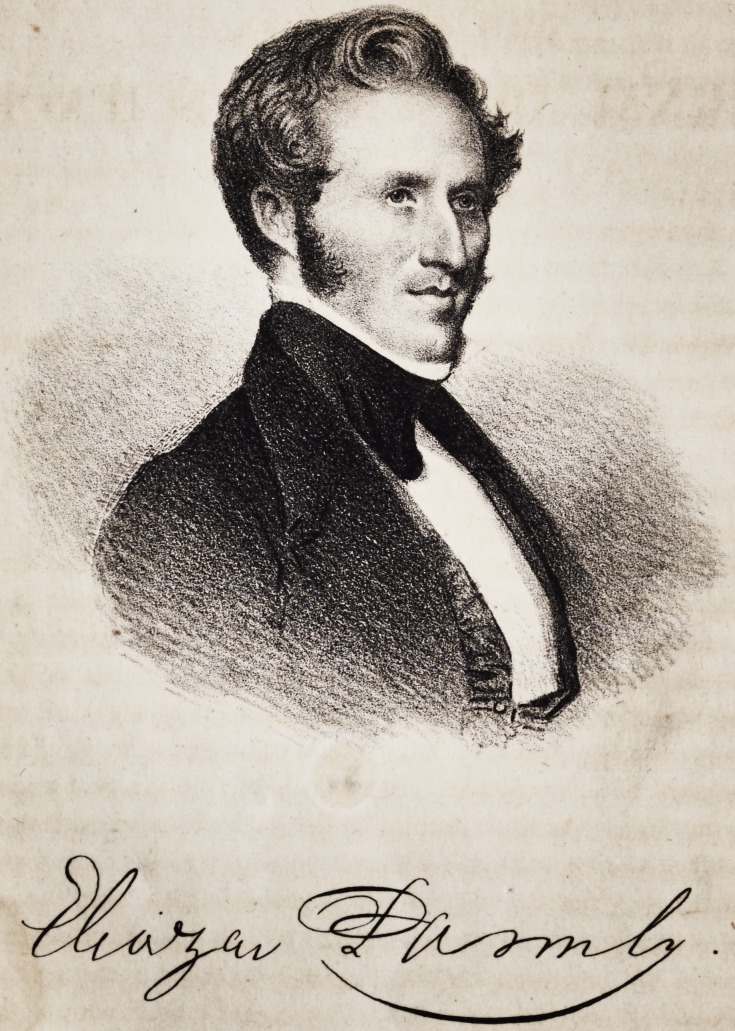# Introductory Address

**Published:** 1842-09

**Authors:** Eleazar Parmly


THE AMERICAN

### JOURNAL OF DENTAL SCIENCE.

VOL. III.] SEPTEMBER, 1842. [NO. 1.

[EXTRACTS from the Transactions of the Third Annual Meeting of the American Society of Dental Surgeons, held in the city of Boston, 19th, 20th and 21st July, 1842.]

### ARTICLE I.

### *Introductory Address.* BY ELEAZAR PARMLY, M. D., D. D. S.

*Mr. President and Gentlemen of the Society of Dental Surgeons:*

As I am about to make some remarks on the professional practice, to which I have devoted wholly and exclusively the last twenty-seven years of my life, perhaps you will not consider the time altogether misspent, if I occupy a few moments in speaking of the difficulties and lack of opportunities for acquiring correct information on any subject connected with practical dental surgery, only a quarter of a century ago. I shall leave it for those who hear me, especially for the younger members of the society, to make a comparison in their own minds between the embarrassments of that period, and the facilities of the present day. As early, I think, as 1812, my brother, older than myself, became acquainted with Dr. Petrie, an English dentist, from whom I believe he obtained his first knowledge of operations on the teeth.

He soon afterwards came to Boston, where he commenced practice, and had the good fortune to become acquainted with the warm-hearted Dr. Randall, to whose friendly interest he was indebted for a handsome letter in testimony of his zeal and ingenuity; which letter he keeps to this day, as a sacred treasure.

To the liberal commendation which the doctor was pleased to bestow upon him in that letter, in addition to the professional information which he frankly communicated to him, my brother was chiefly indebted for his early success.

In 1815, he proceeded to Montreal, where I joined him at the age of eighteen. He employed me at first in carving and manufacturing teeth from the tusk of the sea-horse. On the same year we went together to Quebec, where I saw for the first time a front tooth which had been beautifully stopped with gold by Mr. Waite of London. But I had no idea at the time, how the gold was secured, nor what gave it its firm looking body, and its smooth, solid, and polished surface.

On leaving Quebec for the purpose of a tour through the western states of this union, I did not leave behind me the recollection of the beautiful operation which I had seen, and which even to this day clings vividly to my memory.

While at Pittsburg, I saw a mouth which had been treated by Mr. Koecker, very differently from any I had before seen.

I also saw there some teeth that had been filed by our worthy and esteemed president. These two cases gave me an idea of filing teeth. All that I had before seen where the file had been used, were only separated, as it was then termed with a file; a most destructive practice, particularly with young persons.

In Lexington, Kentucky, I saw the mouths of two young ladies who had been with Mr. Hudson, of Philadelphia, whose stoppings looked like the one I had seen in Quebec.

Up to this time I had done but little, except cleaning, extracting, and making artificial teeth. The few that I had stopped were done with tin-foil, as I had never seen any gold for stopping teeth.

I here obtained some gold coin, took it to a silver-plater, and asked him if he could make it as thin as a leaf of tin-foil which I showed him. He melted, hammered, and rolled the coin, bringing it in his rolling mill to a degree of thinness and pliability, of which I had previously no conception.

This gold I began immediately to use for stopping teeth, and it is now but about three years, since I saw one of the teeth which I then stopped with it in the rudest kind of way.

It was in Lexington that I first saw a mineral tooth, in the mouth of a French gentleman who had but just arrived in this country. This struck me as being a wonderful improvement on the teeth which I was manufacturing from sea-horse and cattle teeth, which I was almost daily in the habit of collecting at the slaughter-houses.

Pursuing my journey to New Orleans, I saw there for the first time in my life, a gold plate, that had been struck on a model in London. All the plates that I had previously seen had been filed and bent into something like the form required. The few operations which I had thus an opportunity of seeing executed in a masterly manner, excited in my mind an ambition which I should like to feel in this life again.

Soon afterwards I embarked for London, with as full and as strong a determination as I think was ever felt by any human being, never again to touch the shores of my native country, until I should know exactly how all these things were done.

My first business after my arrival in London, was to procure all the books on dental surgery that had been published in the English language, but on reading them no one of them told me how to perform in a proper manner any one operation on the teeth, but in the absence of this information, which I so strongly desired, they spoke in strong language of the great benefits resulting from these operations.

I then called upon some of the great men of whom I had heard or read, as being distinguished dentists in the British capital. They all received me politely and conversed with me freely. I speak with great pleasure of the interview which I had with Mr. Gray, of Old Burlington street, who was then, and if alive, is now, one of the first mechanical dentists in the world, besides being a thoroughly educated surgeon.

Mr. Gray dwelt much upon the necessity of becoming first a good mechanic—and upon the utter impossibility of doing justice to the profession or the patient without mechanical knowledge and skill. He himself had gone into practical mechanics thoroughly, and to use his own phrase to me, "could make anything from a needle to an engine." I mention this as conveying a valuable hint to the dental student, and I am anxious to urge it upon his consideration and attention.

After ascertaining how far I could be benefitted by an acquaintance with the London dentists, I went to Paris, and immediately after my arrival there, I called upon some of the most distinguished of the profession. They received me with very great politeness in their well furnished and tastefully decorated antechambers.

When I stated to them the object of my visit to their city, they warmly and cordially wished me success, which of itself was very welcome and agreeable, but still did not supply my wants. I was seeking after higher degrees of knowledge in the dental art.

One day on passing through the Rue Richelieu I saw on a handsome house the name of Maury, dentist to the king's household. Inasmuch as dentists and their works were the chief object of my pursuit, I went in, and Mr. Maury immediately entered the room. I stated to him the object of my visit to Paris, when he instantly led me into his complete cabinet, the first that I had ever entered, and the most perfect that I have ever seen. Here he had every thing at his command, from the earths of which his teeth were composed, to the means of making what in that day were called the most perfect sets of teeth. I at once made arrangements to complete my mechanical studies with him, and when the course was completed, I received from him his testimonial bearing the king's seal.*

I repaired immediately to London where my brother had resided for some time, and established myself in practice.

Here I was in the habit of seeing operations by all the distinguished dentists in London; but Waite's stoppings were the only ones that resembled that which I had seen four years before, and which at that time so completely filled my eye as the perfection of dental practice. Even to this day my mind has not changed in relation to its value. Believing at that time that the difference might be in the gold, I resolved to ascertain who prepared it. I therefore, called at Mr. Waite's house; but as he was absent his brother handed me a part of a leaf of gold, the size of which originally must have equalled that of our largest sheets of tin-foil,

* But for the kind-heartedness of this amiable and excellent man, I might have left Paris without ever seeing a complete cabinet of a dental artist.

and as thick as one of our common sized sheets of gold would be of twenty or twenty-five grains.

After feeling of the gold, I remarked that it seemed soft, yet was very thick; to which he replied—"Yes, it requires great strength to force it into a tooth, but when firmly pressed in, it makes a very solid stopping." I inquired where it could be had, he said "he did not know, but believed his brother got it some where about St. James'."

Most eagerly and faithfully did I look and inquire in vain for it in all the streets, alleys, and courts of St. James; but I have never to this day seen any like it.

I have of late years seen many beautiful operations performed by the London dentists; but in that day Waite stood alone; and I can hardly say more for his practice, than to declare that he was in his day the Hudson of England. He had many eccentricities, and I could relate many anecdotes of him, which I have heard from his patients, but I will not consume your time. I will however mention one because it proves the value of his operations, and the confidence which he felt at an ealy period of his life in his own work.

An elderly gentleman showed me a tooth, which had been stopped by Mr. Waite, more than twenty years before. It was a large and beautiful stopping in a molar of the lower jaw. When the operation was completed, he said Mr. Waite clasped his hands together, and exclaimed, "when you are an old man you will look upon that tooth and say, *blessed be Waite.*" Mr. Waite understood perfectly how to use his hands, and did use them.

I will here take occasion to relate a circumstance to show how coxcombical effeminacy and insignificance will sometimes endeavour to depreciate exalted merit—such things not being altogether unknown in this country. Being out of health one season while residing in London, I was advised to visit Brighton, for the purpose of sea-bathing. While there I called upon the fashionable and celebrated Charles Bew, a kind of hanger-on crony, and dentist of the Prince of Wales. He looked at my teeth and said, "you have been with Waite, sir;" and immediately proceeded to relate an interview which he had recently had with one of his patients.

"My Lady —— was with me (such men never speak of any one, as being a patient, but "my lord," and "my lady,") and while here, she told me that Waite had shown her the knots made on his hands by stopping teeth:" to which he said he replied (and I should think with a most comfortable feeling of self-complacency) "Lord bless your ladyship, those knots were made on Waite's hands by holding on to the straps of his master's carriage."

I hope that we have all learned or shall learn how these callous concretions were made in the hand of the justly celebrated London dentist.

My recollections of the beautiful town of Brighton, the pagoda palace, and romantic cliff of that delightful spot, have always been associated with a feeling of utter contempt for the speech of the celebrated, fashionable, soft and white-handed dentist of the Prince of Wales, afterwards George the Fourth.

I returned to New York, and that city for upwards of twenty years has been the field of my labour.

And now, after having completed twenty-seven years in the uninterrupted and laborious practice of dental surgery, it gives me pleasure to be able to state in a society, composed exclusively of my professional brethren, that during that period, it has been my happiness to witness the efforts of many high-minded and honorable men to advance the interests and usefulness of dental science.

I have hailed with heartfelt satisfaction the numerous and valuable improvements which have resulted to the profession generally, from the labours and investigations of some of the ablest minds that have ever adorned and distinguished any profession.

From the moment when you did me the honour of appointing me to address you on this occasion, it has been my intention to express my opinions in relation to the importance of an association like the American Society of Dental Surgeons; and likewise in as brief a manner as possible to state some of the results of my professional experience.

The necessity for such a society as that which I have the honour to address, has resulted from many important considerations. It is enough to allege on this occasion the facts, that the profession to which we belong has attained to great importance in the estimation of the community: that many individuals of exalted moral worth and high intellectual attainments have made dental surgery the study and business of their lives: that these individuals esteem it to be very desirable that they should be able to avail themselves mutually and reciprocally of the benefit of each other's experience. And furthermore, that by a social and united effort, they may make the attempt to check the progress of empiricism throughout the land, by establishing in the minds of the people throughout this country, the truth, that the members of the association have had their pretensions and qualifications closely scanned, and that they are deserving our approbation and the public confidence. And it will be but a very little while before it will be known who are, and who are not, our associates. Therefore the utmost caution and diligence on our part is called for, and will be necessary in admitting members to our association.

During the period while Randall, Greenwood and Flagg, of Boston; Hudson, Gardette and Koecker, of Philadelphia; Hayden, of Baltimore; and Greenwood and Parkhurst, of New York; not only controlled the practice of this country, but performed with their own hands nearly all the operations in dentistry, which were then demanded of the profession on this side of the Atlantic, there was little need of a society like this to guard the profession from abuses, and protect the public from imposition, because their names were a sure guarantee of capacity and faithfulness in the discharge of their professional duties, according to the measure of the respective capacities of each.

Although they were all alike regarded as men of truth and honesty, yet in their professional practice, they differed widely, as well in regard to the mechanical as to the surgical department of our art. Although mechanical dentistry, denoting the substitution of artificial teeth, in the place of natural ones, has made rapid advances towards perfection within the last few years, the surgical department, embracing all required operations on the living teeth, was mostly, I believe, as well understood, and as perfectly and as thoroughly practised by Hudson, thirty years ago, as by any operators who ever lived either before or since that period.

I now occasionally see operations performed by him, which have remained twenty-five and even thirty years, and which are still as perfect as they were the day they were completed; bearing proofs of as much skill and ingenuity as ever was or ever will be attained by any individual.

We probably are more indebted to his success than to that of any other man for the importance which was attached at that period to operations which were intended to preserve the natural teeth in their natural state.

By the complete success attending the practice of this great man, the public were soon convinced that teeth could be saved, by a proper course of practice; and as a natural consequence of this conviction, persons who felt the need of such aid, repaired in great numbers to those who chanced to call themselves dentists; without stopping to inquire in regard to their individual qualifications to exercise the profession. The disappointment which has followed, has in many instances broken down all distinctions of character and capability, and consequently all dentists are regarded by them without exception or distinction, as adventurers and impostors, preying upon the credulity of the public.

This kind of charlatanism has been and is now practised to such an extent, that I will venture to say that there is more injury done than benefit conferred by operations on the teeth.

Nothing has so degraded the profession and cast so dark and indelible a stain upon its character, as the want of common honesty manifested by most of those who have taken it up, being totally unqualified to discharge with benefit to the community the smallest and least important services required at their hands, unless we except the extraction of a tooth. And yet these men, all unqualified as they are, have rashly undertaken, and do still presumptuously undertake to perform operations which the nicest skill, ingenuity and judgment can barely accomplish.

And while I denounce the impudent impostor, who whether he attempts to operate in either the surgical or mechanical department of our art, brings inevitable disgrace upon himself and the profession, I would not withhold a word of cautionary counsel from some of our ablest men, as well in as out of this society.

It is now more than twenty years since I felt the urgent necessity of making the surgical and mechanical branches of the profession distinct. I regard them as being just as different in practice, as the task of the skilful surgeon in amputating a limb, from that of the artist, who in the exercise of his mechanical ingenuity, contrives joints and springs, which together with other admirable appliances, constitute an artificial one.

Besides, I have never known, with a single exception, any one individual to excel greatly in both these departments of our art. It is here as in Europe the practice of most of our best educated dental surgeons to employ mechanics to do their artificial work, who never see the patients—a practice which never has been found to answer in all cases the valuable purposes contemplated. It is impossible to work to a model as we would work to a mouth; and it is absolutely necessary that the dental artist should have both the mouth and the model before him in every instance.

Therefore while on the one hand I would persuade our well educated men to confine their practice chiefly to operations on the living teeth, for the good of the profession and for the benefit of suffering humanity, I would as earnestly dissuade the mechanical dentists from all attempts at operations in the surgical department; for without the requisite knowledge, and much experience, he will bring disgrace upon himself and the profession, as well as misery upon his unfortunate patients, by dishonest and fatal pretensions to what he does not thoroughly understand.

Such fraudulent proceedings exhibit a want of moral principle, and have done much to destroy that confidence in the profession, which it should be the high ambition of every dental operator to cherish and sustain.

Although the manufacture of artificial teeth is a lucrative employment, yet I hold it to be the duty of every well instructed dentist who excels in the surgical department of his art, to abandon the making of artificial teeth as far as practicable, and especially when his time can be employed to better purpose. If the time consumed in manufacturing a set of artificial teeth were devoted to the preservation of natural ones, the accomplished dental surgeon would secure to his patients a greater amount of comfort than ever was conferred by artificial substitutes, even by the best that ever were constructed.

The much beloved and highly respected Hudson would have transmitted to this day no proof of his masterly skill, had his life been spent in manufacturing such short livid evidences of merit as artificial teeth.

Yet there is another class of dentists who greatly excel in this kind of manufacture, and who arrange and adapt these valuable contrivances in such a manner, as to deserve the very handsome remuneration which they obtain; besides securing to themselves the respect and gratitude of thousands, for thus ingeniously repairing the losses occasioned by accident and disease.

If in our efforts to improve dental surgery we can effect a division of labour among its professors in the large cities, so that each individual will be employed chiefly in that branch in which he particularly excels, individual merit will be soon known, and should it ever fortunately happen in the numerous changes in human society, that the public shall make a distinction between merit and imposture, then may quackery and all its attendant evils meet their recompense.

I can conceive of nothing but the accession of clever men to our ranks, men who shall be distinguished as well for truth and integrity, as for professional abilities, that ever can correct the abuses of which ourselves and the community so justly complain. Whatever tends to bring about objects so much to be desired, will in after ages, be regarded by the profession as the strongest evidence we could transmit to them, of our desire to elevate our art and extend its usefulness.

The community, moreover, in the midst of which we live, will regard us with feelings of gratitude and respect, for having done something to relieve them from the imposition and abuse to which they have been so long and so unfortunately exposed.

There is no operation in surgery which requires greater concentration of thought, or greater delicacy of execution, than do operations in certain stages of dental disease, and on certain kinds of teeth; nor is there any branch of mechanical industry which requires more perfect accuracy of execution, than is sometimes required in overcoming difficulties in constructing and adapting artificial teeth.

If the public rightly understood these distinctions, and if they who understood would be governed by them, we should be rid of the swarm of swindlers, and impostors, that so encumber and degrade the dental profession.

It has been considered necessary in this country, although I do not know any good reason for it, that dentists should uniformly embrace in their practice both the surgical and mechanical branches. Hence it frequently happens that the man who is really a good mechanic, understanding well how to handle inanimate matter, undertakes operations on the living teeth, without knowing that they are highly organized bodies; endowed with a living principle, possessing nerves and blood-vessels, and therefore are of an exceedingly delicate structure. Such a man by his harsh and bungling treatment destroys their vitality and thereby brings himself into an attitude worse than that of an ordinary robber, entailing sorrow and suffering on his deluded patients.

So also the well educated surgeon for no other reason than because his neighbour does the same thing, begins to tamper and meddle with artificial teeth; and really, some of the roughest pieces of "botch work" that I have ever seen, have come from the hands of men well educated in medical and surgical science.

When such things are done by such men, we are compelled to ascribe it either to dishonesty or folly; to the former, if a high price has been charged for such bungling productions; to the latter, if gratuitously performed.

While this state of things exist, which is to be seriously lamented, the utmost confusion will distract the public mind in regard to dental practice—merit and imposture will be equally rewarded; the profession will be slandered and the public abused.

It is a well known fact, that until within a few years, and even to this day, to a limited extent, as well in country towns as in our largest cities, the family doctor was physician, surgeon, aurist, oculist, dentist and apothecary.

Look now at these different departments of the healing art, and observe the incalculable advantages of a division of labor among sundry individuals, each educated to his particular branch. The community at large as well as the medical profession, have benefitted largely by this change.

And similar advantages, I am convinced, would accrue to the public and to ourselves, by a division of labour in dental practice. As a safeguard to the community and to the profession, it seems to be as much needed as in the other branches of the healing art.

From impostors and swindlers, men without truth, honor, or moral obligation, we can expect no reform. They will be impostors and swindlers still—for they are impelled by the instinctive depravity of their natures: but from the gentlemen composing this society, and from some few others who are not yet united with us, but whose names would do honor to any society or profession, both ourselves and the public have much to expect in this great work of reformation.

The great object contemplated in our social union from the outset, was to correct these abuses; and if we persevere in our noble undertaking, performing faithfully and honestly our social and professional duties, it will be but a short time before our labours will be appreciated—the profession improved, and the public protected.

I would here take occasion to remark on the subject of taking dental students, it has been made a point with some not to take them until they should have fully completed their medical and surgical studies. This idea, based as it is upon the desire to advance the interests of the profession, by bringing into its ranks well educated men, I am now persuaded is entirely a mistake. The hand must be first educated—I never knew a man whose early life had been mainly devoted to study, and who took up the profession of dentistry after the age of twenty or twenty-five, who became a good mechanic. But we have known men who were bred in early life to mechanical labor, who have not only become the ablest surgeon dentists, but have in many instances become the most accomplished and profound scholars, and the ablest as well as the most distinguished men in the learned professions.

It appears then that it is only in youth that the mechanical arts are most perfectly acquired—and it deserves to be most particularly noted, that all operations in surgery require a practical or mechanical hand, what must be acquired in youth by exercising it in some way or other.

The study of the sciences which require much serious thought, judgment, and deliberation, may be commenced, as has been often proved, at a late period of life, and prosecuted with the most perfect success.

I would therefore most earnestly recommend to all young men preparing for the profession, to employ a portion of their time while young, or while pursuing their studies, in some nice mechanical work, for although an acquaintance with medical and surgical science is absolutely necessary, and cannot be dispensed with by the successful practitioner, I do positively assert as the result of much observation, and I assert it with the strongest emphasis too, that it is only when the qualifications of the skilful surgeon are super-added to those of the ingenious and accomplished practical mechanic, that a man can enter into both departments of our profession, and practice successfully as a surgical and mechanical dentist.

Many persons repose great confidence in men who have once been in the medical practice, and who have exchanged the practice of medicine for that of dentistry. But I wish the community to understand, that although a good mechanic may become a great physician, I do not believe that there is a single physician in the United States, twenty five years of age, who could become a nice, ingenious, and expert mechanic, such as a dentist must be to be successful, unless he had used his hands in labour while young.

I have dwelt on this subject perhaps too long, but I have been led to do so from having for twenty-seven years witnessed the misery and deformity occasioned by bungling and abominable fixtures in the mouth, constructed without the least pretension to mechanical skill.

When health and comfort on the one hand, or disease and misery on the other, must of necessity be the alternative, let our young dentists feel a pride in becoming in the mechanical department of our art, the first mechanics in the land. The profession demands it; and even if they have not the love of that profession at heart, let the handsome manner in which the public are willing to pay for such services, encourage them to do it.

Thus three important objects will be gained—that of the operator in securing reputation, usefulness, and emolument—that of the profession generally, in finding their art honoured among men—that of the community, in being faithfully served.

Let me add while on this part of the subject, that I sincerely deprecate and utterly condemn the practice of some men of our profession, for taking students for a few weeks or months, and instructing them for a few hundred dollars.

I unhesitatingly say, that no man feeling the heavy responsibilities of a dentist, and what the public have a right to expect from those whom they entrust with their teeth, will ever send abroad students thus educated in the dental art.

It being now admitted on all hands that teeth can be saved by proper operations, the public are ready to inquire, "How are we to avail ourselves of the benefits of these operations ?" The answer is simple—employ skilful men ! And dentists too inquire, "how are we to become skilful ?" The answer to them is, apply yourselves to practical study under the instruction of able men, taking them and them alone as your guides and examples.

It is an easy matter to describe the best mode of performing all operations on the teeth ; but it must be a hard matter to describe an operation in such a way, as to enable another to take the description and go away and perform that operation.

An ingenious jeweller possessing the greatest power of description ever inherited by man, might call around him a hundred of the most refined and best cultivated intellects that can be found in the learned professions, and describe to them the best mode of cutting a diamond and setting it in a ring, and I will venture to say that not one of the hundred would be able to make the ring and set the diamond, although one of the apprentice boys who did not understand the meaning of half the words used in the description of the process, would at once take the materials in a rough state, and perform the task, not because his head alone, but because his hands also possessed the necessary cunning. And yet the difficulty of preparing the diamond and sitting it on the ring, bears no just comparison with the difficulty of securing the gold in a tooth in certain cases. Hence the indispensable necessity of combining mechanical ingenuity and skill with correct professional knowledge.

In general terms I would say then, that in stopping teeth, it is essential in all cases that the cavity which receives the gold, should be made larger within, than at the orifice or external opening, and then that the gold should be introduced with the utmost niceness and care, being made as solid by compression as the nature of the case will admit.

If the gold is good, and I am sorry to say that we are not always well served, even by the best manufacturers, the body of the stopping will be firm, and the surface will receive a polish.

When a tooth has been thus stopped, the durability of the stopping will depend on several contingencies.

First, on the preparation of the cavity; secondly, in the ingenuity with which the gold is introduced and compressed; and thirdly, on the character and strength of the tooth, whether it is or is not disposed to crumble around the stopping, thereby weakening its hold.

Care should be taken to go no deeper into the tooth than is absolutely necessary to remove the decay and form a cavity.

No man can form a cavity properly without proper excavating instruments.

I have a set of instruments purchased from the widow of a certain medical gentleman in New York, who came out largely a few years ago as a dentist. With these instruments I will defy the most expert operator and ingenious dentist in the world to stop a front tooth, without destroying the nerve in the operation; and it is by such men, and such instruments, that we see so many discoloured and black front teeth in the mouths of young people, who have had them thus stopped.

It is a common practice when the front teeth are decayed, to file a space between them wide enough to enable the operator to get at them to fill the cavity. This method in the hands of a skilful man is generally a safe one, but for ten years past I have in many instances abandoned it, and particularly where I could do so with young people, and instead of filing, I have separated the teeth by forcing between them slips of India rubber or thin wedges of wood, which by its expansion, cause the teeth to recede without any injury. Pursuing this course for a few days, enlarging the wedges from day to day the cavity may be stopped. Whereupon the teeth will immediately fall back into their original position.

When this is done there is not the same danger of wounding the nerve as when the file has been used, for the reason that sometimes we are compelled to go beyond the depth of the decay, in order to get a cavity deep enough to hold a stopping, and in thus making it deep enough, in many instances, the nerve is touched, and the vitality of the tooth consequently destroyed, which would not have been the case if the plan of wedging had been adopted, for all that the file removed would have been left to form a secure hold or support for the gold.

Gold is the only substance that I recommend for a permanent stopping.

Although I sometimes use tin-foil it is only where the tooth will not bear the force necessary to secure a gold stopping. In such cases and also in a few peculiarly shaped cavities, it being softer it answers a valuable purpose for a time, and is renewed as occasion may require.

I condemn in the strongest terms, succedaneum, mineral paste, diamond cement, and every other vile composition in which there is a mixture of quicksilver.

Some unprincipled men have gone so far as to say I use it in order to recommend it in their practice. Patients who have had their teeth stopped with it, on being inquired of who stopped them, have said that I did it, for which I can see no other reason than that they are ashamed to confess in whose hands they had the folly to place themselves, and prefer telling a wicked falsehood implicating another, rather than admit that they were among the "humbugged" of the day.*

Some who use these preparations say that there is no mercury in them. I would tell these same honest gentlemen who pretend to have large establishments in the principal cities of Europe, that

*It seems that patients who have had their teeth filled with it have not only made such statements here, but the same has been stated abroad, as will be seen from the following extract from a letter written to me by the highly distinguished Charles Bromley, of England. "You use the cement occasionally I know, for I saw a patient of yours only a week or ten days ago who had some teeth stopped with it—you are aware that it frequently destroys the vitality of the teeth by its harshness, weight, and irritating properties." I would here reply to that portion of Mr. B's letter, and say, that I am fully aware of its destructive properties and its total unfitness for stopping teeth. I once prepared some of it and filled a dead tooth with it for Mr. N. of New York, and he is the only person in the world that can exhibit a tooth ever touched by me with it—having proved its deleterious effects, I uniformly condemn it and have condemned it for many years.

there is mercury in their stoppings, unless they are some of them very different from those I have repeatedly taken from the mouths of their deluded patients.

I would gladly have spent more time in speaking of practical and operative dental surgery, but the time allowed me for this address is nearly expired, I will therefore only occupy a few minutes longer while I read the following lines.

I here would ask a further pause,
While I in loosely measured rhymes,
Enforce the objects of a cause,
Descended from remotest times.

Far distant lands beyond the sea,
In dental science oft have found
A charm for mortal misery,
A balm for many a fest'ring wound.

Britain can boast of noble minds,
Whose lives and energies were spent
To raise the veil which meanness binds
Around the souls on myst'ry bent.

Hunter and Fox deserves our praise,
Who gave to dentistry their fame,
Bell, Hare, and Nasmyth, in our day,
Have added honors to the name.

In practice too, much has been done,
Evincing skill exceeding great,
By Edmonds, Newton, Parkinson,
Cartwright, and Bromly, Gray and Waite.

And France has nobly done her part,
In bringing from the earth beneath,
Its treasures, to enrich our art,
In ample stores of mineral teeth.

And in the list of authors too,
That have advanced the science far
We find Fouchard, Le Maire, Foucou,
Maury, Le Baume, and Delabarre.

Old Spain has brought into the field
Her Garnott his part to take,
Ireland to none the palm will yield,
And points to Hudson and to Blake.

In Germany, whose pride records
  The fame of many a knightly chief,
Ere she to ploughshares beat her swords,
  Koecker stands forth in bold relief.

Can we sit still and with content
  Beneath the skirts of genius lurk,
And leave no trace to represent
  Our part in all this glorious work.

Has not this favoured land of ours
  Brought forth its names of brilliant worth,
For active and inventive powers,
  Equal to any known on earth.

In intellect may they not vie
  With mightiest minds of other lands;
One brought the lightning from the sky,
  While others broke oppression's bands.

Her warriors look throughout the world
  On every sea, and land, and coast,
No banner proud was e'er unfurled
  By braver men than she can boast.

Her statesmen—search the earth around,
  Both in the past and present day;
And where can nobler men be found,
  Than Webster, Washington and Clay.

Her artists, too, in this, our clime,
  Now rank among the very best;
Better not known in any time,
  Than Inman, Trumbull, Alston and West.

Her poetry triumphant stands
  Amid the blaze of Europe's light,
In Dana, Pierpont, Drake, and Sands,
  In Halleck, Bryant, Dawes, and Dwight.

Among high authors rich and chaste,
  Who e'er did purer thoughts bestow,
Than are in Channing's works embraced,
  Or from the pen of Irving flow.

In surgery and medicine,
  Has it not been our happy lot,
The praise of all the world to win,
  In Warren, Physick, Rush, and Mott.

Ambition points to these bright stars
  So dazzling in the world of mind,
In art—to steamers, ships, and cars,
  That leave all others far behind.

May this society, our pride,
  So fondly cherished in our hearts,
Here after flourish side by side,
  With honoured sciences and arts.

May we not in our native land,
  In after days receive, perchance,
Some praise for binding in one band,
  All who would see our cause advance.

Can we abandon or neglect
  An object when the aim's so good;
Ah no! the public will respect
  Our labours when they 're understood.

Some generous minds have lent their aid
  To help the noble enterprise;
While others seem to be afraid,
  And shrink from what before us lies.

That is to build both firm and strong
  An institution, gathering fame,
That all who may to it belong
  Shall honoured feel to wear its name.

Dr. Parmly having finished his address, Professor Hayden delivered a lecture on the Diseases of the Antrum Highmorianum, the publication of which, however, among the transactions of the society, he declined, on the ground that he intended to publish it in a treatise on Odontology, which he had in the course of preparation.

Eleazar Parmly.

Published by direction of the American Society of Dental Surgeon's for the, Journal, and Library of Dental Science.

## Figures and Tables

**Figure f1:**